# Towards P2X4 Positron Emission Tomography Tracing

**DOI:** 10.1021/acs.jmedchem.5c00772

**Published:** 2025-03-27

**Authors:** Stephanie Federico

**Affiliations:** Department of Chemical and Pharmaceutical Chemistry, University of Trieste, Via Licio Giorgieri 1, 34127 Trieste, Italy

## Abstract

The P2X4 receptor is involved in immunological and inflammatory processes and potent antagonists are potentially useful for therapeutic, investigational, and diagnostic purposes. This Viewpoint summarizes the discovery of potent and selective P2X4 antagonists that bring researchers closer to obtaining valuable PET tracers for studying the P2X4 receptor.

The P2X4 receptor (P2X4R) is highly expressed on immune cells and neurons, and it is involved in cancer, neuropathic pain, and neuroinflammatory disorders like neurodegenerative diseases. Consequently, P2X4R antagonists hold significant therapeutic potential; however, their development remains limited, with only a few showing moderate efficacy. Moreover, given the involvement of P2X4R in various CNS disorders and cancer, Positron Emission Tomography (PET) tracers could facilitate further investigation of this target. For these reasons, the development of potent and selective P2X4R antagonists is currently a highly active area of research, as evidenced by the growing number of publications, including the Featured Article by Erlitz et al., featured in this issue.^1^

Purinergic receptors play a key role in a plethora of patho-physiological conditions. They are divided into P1 (adenosine) and P2 receptors, with P2 further subdivided into P2X and P2Y receptors. P2X receptors are ligand-gated cation channels activated by ATP. P2X4R, a homotrimeric receptor, is highly expressed on immune cells and neurons, and its surface expression increases in neuroinflammatory conditions like neuropathic pain and multiple sclerosis.^2^ P2X4Rs have been found to be involved also in cancer progression and agressiveness.^3^ Despite its importance, there is a limited number of P2X4R antagonists reported in the literature, with few showing high efficacy and/or selectivity. Among approved drugs, Duloxetine (**1**) and Paroxetine (**2**) (Figure 1), which are currently used as antidepressants due to inhibition of monoamines reuptake, present an off-target antagonistic activity on P2X4R, in fact, they reverse tactile allodynia. Instead, most representative P2X4R antagonists are reported in Figure 2.^2^

**Figure 1:**
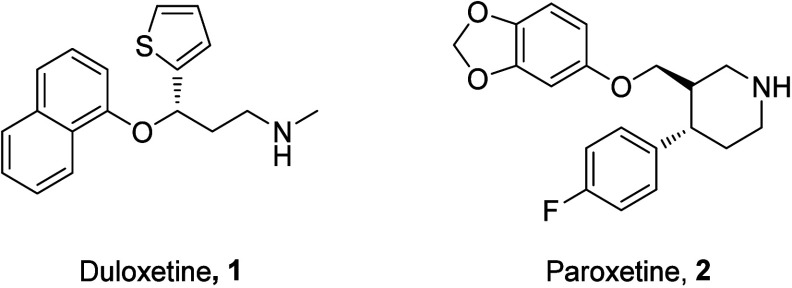
Antidepressants with off-target antagonism on P2X4R.

**Figure 2:**
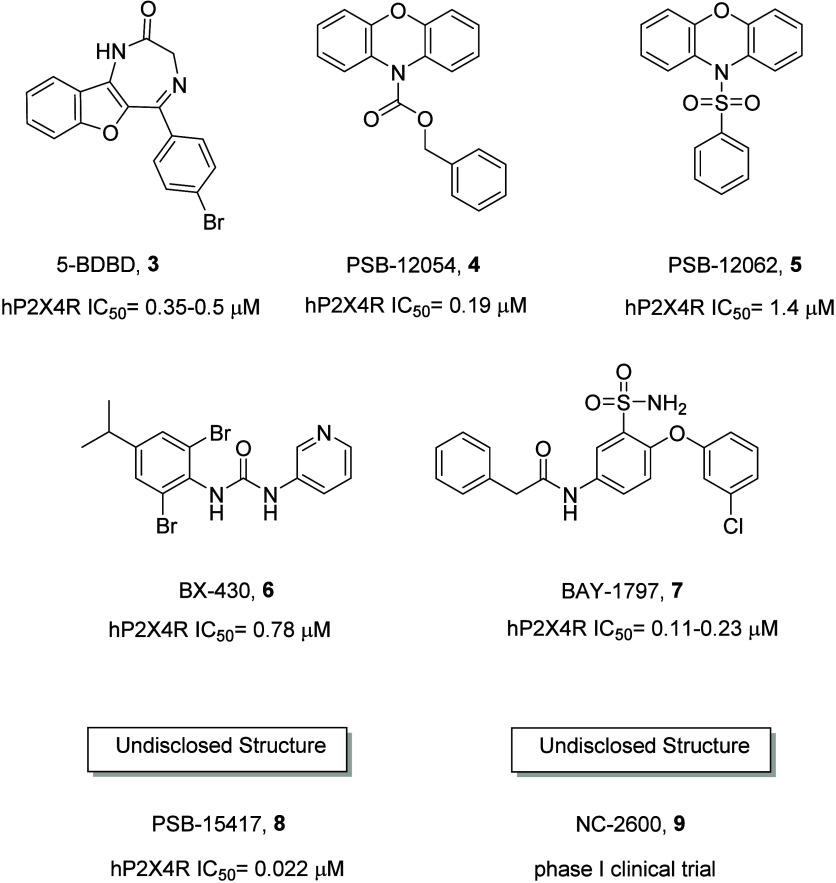
Representative P2X4 antagonists.

It is quite evident that there is not a big variety in chemotypes reported as P2X4 allosteric antagonists. 5-BDBD’s (**3**) central core is a 1,3-dihydro-2H-benzofuro[3,2-*e*]-1,4-diazepin-2-one with a low water solubility. The phenoxazine nucleus is, instead, present in the allosteric P2X4 receptor antagonists PSB-12054 (**4**) and PSB-12062 (**5**), where the second, even if less potent, it is more water-soluble than the first. Then, during a screening campaign, urea derivative BX430 (**6**) has been found to behave as a submicromolar allosteric antagonist at the hP2X4 receptor. The last chemotype reported in Figure 2 is represented by the sulfonamide of BAY-1797 (**7**), which, counterpartying its high water solubility, does not permeate the blood–brain barrier. In addition, in the literature, two P2X4 antagonists, PSB-15417 (**8**) and NC-2600 (**9**), which structures have not been disclosed, are reported to be potent and selective. In particular, PSB-15417 (**8**) is reported to be brain-penetrant and showed significant activity in animal models of neuropathic pain, while NC-2600 (**9**) actually concluded a phase 1 clinical trial. Initially indicated for neuropathic pain, chronic cough has been then added to the primary indications of this compound.

A promising class of P2X4 allosteric antagonists explored by different research groups, including Erlitz et al., is represented by 1,4-naphthodiazepinediones (Figure 3).^1^ The parent compound of this class is NP-1815-PX (**10**), a molecule developed by Nippon Chemiphar Co. and Kyushu University (the same of NC-2600), that was selective for human P2X4R versus other P2XRs tested, with high potency and water solubility. Jacobson and co-workers developed a series investigating substitutions on the naphthyl rings or replacement of the phenyl ring at the 5 position of the diazepine ring with pyridines. The work led to the discovery of compound **11**,^4^ where the replacement of the 5-phenyl ring of **10** with a pyridine increased affinity of 3-fold at the P2X4R with a similar selectivity profile, data reported in Figure 3 have been obtained with the same assay for these two compounds. Nippon Chemiphar Co. claimed also the potent antagonist compounds **12** and **13**, where the phenyl at the N5 position has been substituted at the *para* position instead of the *meta* position like in compounds **10**–**11**.^1^

**Figure 3:**
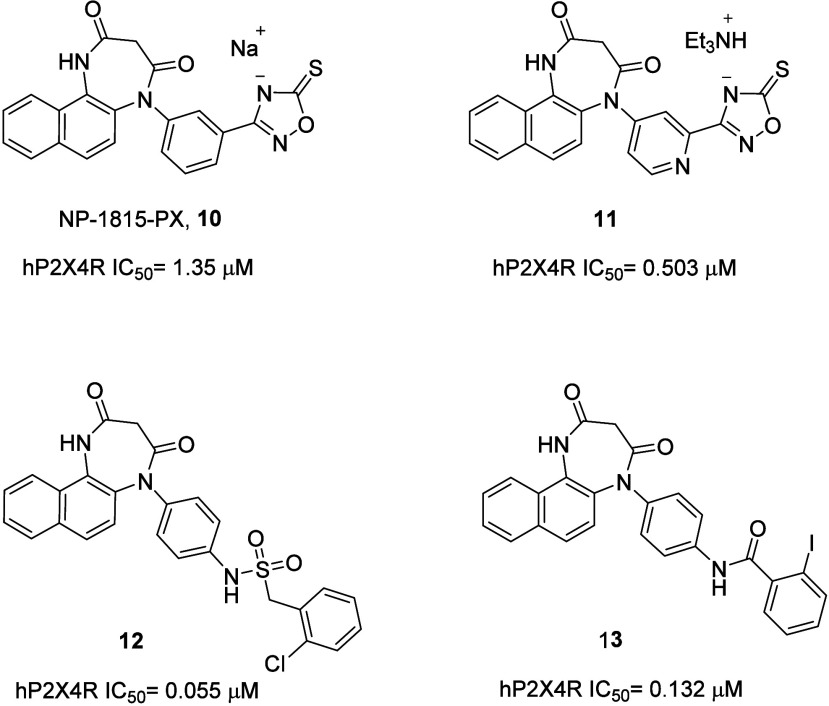
Representative 1,4-naphthodiazepinediones as P2X4 antagonists.

Erlitz et al. identified compounds **12**–**13** as promising lead structures for a structure–activity relationship (SAR) study, especially because the presence of the amino group allows one to perform a late-stage diversification, thus reaching a huge number of diverse molecules in a short time. In fact, the authors developed 67 1,4-naphthodiazepinedione-based P2X4 receptor antagonists, exploring different analogs of compounds **12**–**13** possessing the general structures I–III reported in Figure 4. Investigation focused on the distal phenyl ring, where different substitution have been introduces both in the amido (general structure I) and sulfonamidomethyl (general structure II) series. In addition, also the removal of the methylene bridge of the general structure II has been explored (general structure III). This exploration discovered several nanomolar antagonists, SAR shows that methylene bridge between the amide and the aromatic ring favors H-bonds formation by solfonamido group, while a substituent at the 2 position is crucial to constrain free rotation of the aryl ring, stabilizing it inside the binding pocket. Substitution at the 4 position is less important, but a fluorine perfectly fits in the binding site. Fluorine has been largely introduced in these compounds, because authors would like to develop PET tracers. Some potent compounds have been further characterized for some DMPK properties, e.g. LogD7.4, Plasma Protein Binding (PPB), and Mouse Liver Microsomes Stability (MLM). This is important both for therapeutic and PET tracer development purposes. Compounds **14** and **15** (Figure 4) have been finally selected for *in vitro* evaluation and for the development of PET tracers. It is important to note that methylene bridge in compound **14** demonstrated to be a point of metabolic instability (MLM stability after 90 min is of 64%), while the corresponding arylsulfonamido derivative **15** has higher stability in mouse microsomes.

**Figure 4:**
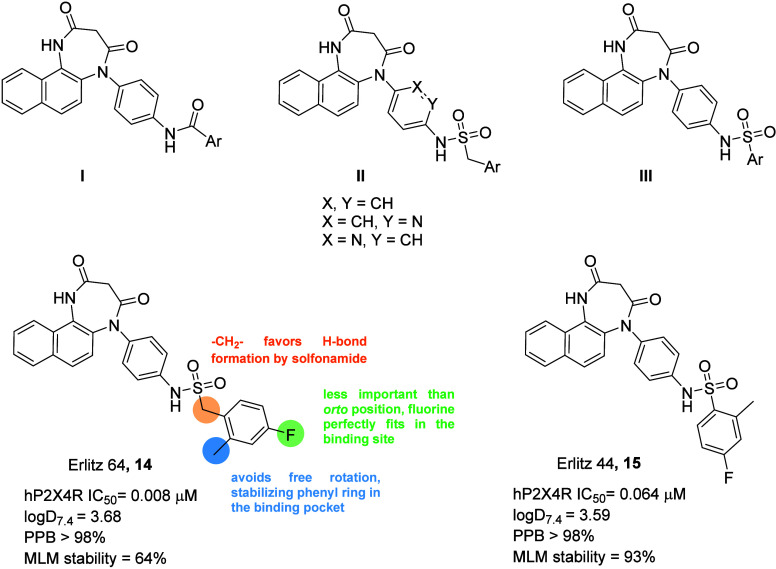
General structures of developed compounds by Erlitz et al. and most representative compounds of the series **14** and **15**. SAR results have been summarized on compound **14** structure.

The therapeutic potential of most promising compounds has been preliminary evaluated assessing their ability to inhibit interleukin-1β release by NLRP3 Inflammasome in macrophages. Erlitz 64 (**14**) and Erlitz 44 (**15**) proved to be able to block NLRP3 inflammasome activation. According with the scope of this work, these compounds were easily obtainable in the ^18^F-radiolabeled form, thanks to the easily obtainment of the boronic acid precursors that were subjected to a copper-catalyzed radiofluorination with [^18^F]KF. Despite their good lipophilicity, radioligands Erlitz [^18^F]64 (**16**) and Erlitz [^18^F]44 (**17**) behave in a different, but rapid, *in vivo* metabolism and displayed almost no blood–brain barrier (BBB) permeation, thus limiting their PET tracer potential without further structural optimization. Even if a PET tracer for P2X4s is still unavailable, this work represents the most advancement in the field, where a complete *in vivo* study on the radiolabeled compounds has been carried out. In fact, the only other published tentative has been made on 5-BDBD (**3**), where both ^11^C and ^18^F analogs (**18**–**19**) have been synthesized, but only binding experiments has been reported to support their possible use as PET tracers (Figure 5).^5^ The obtained insights in the SAR of 1,4-naphthodiazepinedione-based P2X4 receptor antagonists, combined with *in vitro* and *in vivo* pharmacokinetic data, will probably allow researchers to early overcome the metabolism issues encountered, thus we could have the first P2X4R PET tracer soon.

**Figure 5:**
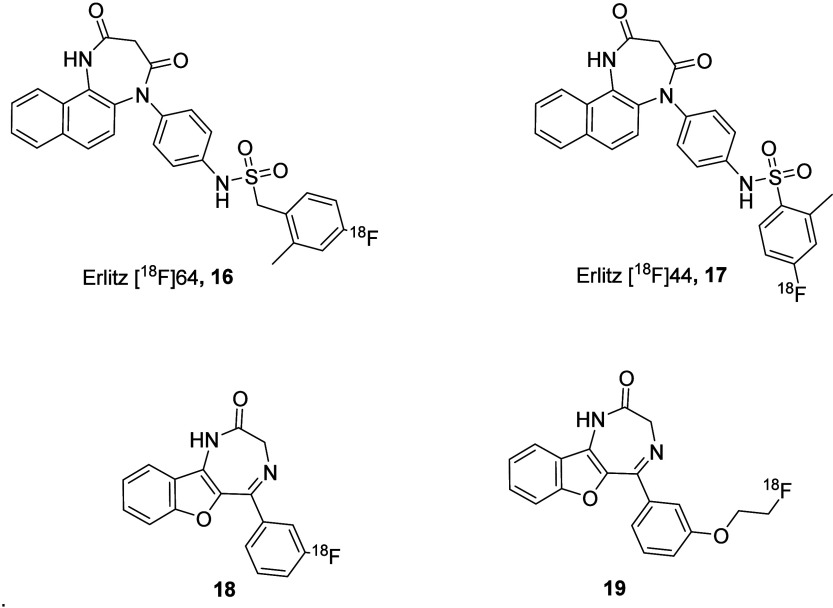
P2X4 ^18^F-ligands developed as PET radiotracers.

It is important to highlight that an important achievement has been recently obtained in the field of P2X4R. Until now, only apo and agonist bound structures were available, impeding to obtain a reliable model of the receptor for the study of allosteric antagonists. In 2023, the cryo-EM structure of a zfP2X4 bound to allosteric inhibitor **7** has been solved (PDB: 8IGH).^6^ Thus, Erlitz et al. built a homology model of hP2X4, based on this structure to perform both docking studies and molecular dynamics simulations. The authors have obtained important insights on the key interactions in the allosteric binding pocket, paving the way to develop a more efficient structure-based design. All compounds docked in a similar way at hP2X4, with the naphthodiazepinedione ring directed to the pore, and the substituted aryl group located in the allosteric binding site, extending toward the outside.

Very recently, the Muller group published an article highlighting species differences of known P2X4 receptor modulators.^7^ This further underscores P2X4R as a highly relevant topic in the purinergic field. The authors observed significant species differences for the allosteric antagonist 5-BDBD (**3**), one of the most widely used pharmacological tools for P2X4R, which shares structural similarities with compounds reported in the featured article by Erlitz et al. Since animal models remain indispensable in drug research and development, serving as the gold standard before clinical trials, understanding species differences is crucial for properly designing and interpreting *in vivo* studies.

In conclusion, Erlitz et al. pursued a dual objective: developing compounds for both therapeutic use and PET imaging. Notably, their work highlights the challenges in PET tracer development, providing valuable insights for this and other research groups striving for success in this field.
